# The in vitro modulation of steroidogenesis by inflammatory cytokines and insulin in TM3 Leydig cells

**DOI:** 10.1186/s12958-018-0341-2

**Published:** 2018-03-22

**Authors:** Kristian Leisegang, Ralf Henkel

**Affiliations:** 1 School of Natural Medicine, University of the Western Cape, Private Bag X17, Bellville, 7535 South Africa; 2 Department of Medical Biosciences, University of the Western Cape, Private Bag X17, Bellville, 7535 South Africa

**Keywords:** Steroidogenesis, Testosterone, Progesterone, Leydig cells, Cytokines, TNFα, IL1β, IL6, IL8, Insulin

## Abstract

**Background:**

Cytokines and hormones, including insulin, are known to modulate the hypothalamic-pituitary-testes axis and steroidogenesis, both centrally and peripherally. In the context of chronic inflammation and hyperinsulinaemia mediating male hypogonadism associated with obesity, metabolic syndrome and type 2 diabetes mellitus, these mechanisms are poorly understood and the impact of cytokines and insulin on Leydig cell steroidogenesis has not been fully elicited. This study aimed to further investigate the in vitro impact of TNFα, IL1ß, IL6, IL8 and insulin on Leydig cell function and steroidogenesis.

**Methods:**

hCG-stimulated TM3 Leydig cells were exposed to various concentrations of TNFα, IL1ß, IL6, IL8 (100 ng/ml, 10 ng/ml, 1 ng/ml and 0.1 ng/ml) and insulin (10 ng/ml, 1 ng/ml, 0.1 ng/ml and 0.01 ng/ml) in optimal cell culture conditions over 48 h. Cell viability (XTT) and testosterone and progesterone concentrations (ELISA) were assessed using standardised laboratory techniques.

**Results:**

TNFα significantly decreased cell viability and progesterone and testosterone concentrations in a dose-dependent relationship. IL1ß and IL6 had a subtle but significant negative effect on cell viability and testosterone concentrations, with a marked significant decrease in progesterone concentration at all concentrations investigated. IL8 showed an increase in cell viability, with no significant effect on testosterone concentrations alongside a significant decrease in progesterone concentrations. Insulin significantly increased cell viability and testosterone concentrations in a dose dependent relationship, but interestingly significantly decreased progesterone concentrations.

**Conclusions:**

The inflammatory cytokines TNFα, IL1β and IL6 cause a dose dependent decline in steroidogenesis in TM3 Leydig cells. These results suggest that chronic inflammation may downregulate steroidogenesis in males via direct modulation of Leydig cell function. However, IL8 may stimulate TM3 Leydig cell growth. Insulin is associated with a dose-dependent increase in testosterone synthesis, with a significant decline in progesterone synthesis. With the phenomenon of insulin resistance, the literature is unclear on the potential role of hyperinsulinaemia in steroidogenesis. Further studies are warranted in order to fully elicit the molecular mechanisms and interactions of these molecules on male steroidogenesis.

## Background

Evidence suggests that immune regulating cytokines, including TNFα [1], IL1 [2] and IL6 [3], and hormones such as insulin [4], modulate the hypothalamic-pituitary-testes (HPT) axis. These effects are mediated centrally via modulating GnRH and LH, and peripherally via direct action on Leydig cells and Sertoli cells [1, 3, 5]. Optimal Leydig cell function within the HPT context is critical for steroidogenesis cascades and primarily testosterone production, a central hormone for male fertility and general male health and well-being [5, 6].

Testosterone is a steroid hormone primarily produced by Leydig cells in the interstitial space of the testes [7]. The role of testosterone in male fertility is well defined, particularly via action on Sertoli cells to promote spermatogenesis [8]. Additional roles for testosterone include muscle formation, body mass composition and fat regulation, bone mineralisation and cognitive functions [7].

Male hypogonadism, characterised by testosterone deficiency and relevant clinical features, affects approximately 6% of males with an increasing incidence and prevalence globally in recent years [5]. Although an uncommon underlying cause of male infertility, serum total and free testosterone concentration should be considered in the assessment of male infertility cases [7]. Clinical features of hypogonadism include sexual dysfunction, reduced muscle strength, increased abdominal adiposity, sleep disturbance and psychological disturbances, and co-morbidities such as dyslipidaemia, hypertension and hyperglycaemia [5]. There are numerous potential causes of male hypogonadism, which can be further classified as testicular failure (primary) or of hypothalamic or pituitary origin (secondary; hypogonadotropic) hypogonadism [7]. Although serious acute and chronic inflammatory pathology is associated with primary gonadal failure [5, 9], obesity and related co-morbidities (e.g. metabolic syndrome and type 2 diabetes mellitus) are considered to be the single most common cause of male hypogonadism, affecting more than 50% of obese males [10, 11].

Traditionally, the steroid hormone progesterone has been viewed as an unimportant precursor hormone in male physiology. However, progesterone is an important modulator of male endocrine and reproductive function [12, 13]. Progesterone in men is synthesised primarily in the adrenal glands, with some production in the testes, and is an essential precursor for all steroid hormones, including testosterone. Progesterone further regulates the hypothalamus and pituitary gland in the synthesis of GnRH and gonadotropins (LH & FSH), respectively, and regulates sexual behaviour in the brain. Evidence also suggests that progesterone has various modulating functions in the central nervous system in males, and therefore affects mood, behaviour and cognitive functions [12].

Although numerous cytokines and insulin are known to modulate the HPT axis, the role of these polypeptides on modulation of male reproduction and steroidogenesis has not been fully elicited and requires further investigation [1, 3]. Inflammatory cytokines are increasingly associated with male hypogonadism, and a direct causal association is postulated [5]. Furthermore, this is relevant in light of the interaction between chronic inflammation, hyperinsulinaemia and male hypogonadism that may mediate obesity, metabolic syndrome and type 2 diabetes mellitus related male reproductive dysfunction and hypogonadism [14–16]. Sex steroids and inflammatory biomarkers are known to be associated with the pathogenesis of various chronic and degenerative diseases in men, although these relationships remain poorly understood [17].

Within this context, the impact and associated mechanisms of action of inflammatory cytokines and insulin on steroidogenesis is required. As testosterone synthesis is predominantly in Leydig cells of males [18], the aim of this study was to investigate the impact of inflammatory cytokines (TNFα, IL1β, IL6 and IL8) and insulin on steroidogenesis using a TM3 mouse Leydig cell line. Results of this study will be of interest to further understand how steroidogenesis may be modulated in chronic inflammatory diseases and obesity related metabolic complications.

## Methods

A TM3 Leydig cell culture model was used in order to investigate the effect of insulin and cytokines on cell viability and steroid hormone (testosterone and progesterone) production. TM3 cells are Leydig cell lines derived from 11 – 13d mouse testes (ATCC No CRL – 1714, Manassas, VA, USA). This was an in vitro experimental study comparing various concentrations of experimental exposure to a control group.

In brief, for each set of experiments, cells were seeded and incubated under standard laboratory conditions for 48 h. Following this, the cells were then exposed to various concentrations of TNFα, IL1β, IL6, IL8 (0.1, 1, 10, 100 ng/ml) and insulin (0.01, 0.1, 1, 10 ng/ml) in hCG-enriched (25 mIU/ml) media for a further 48 h. These experiments were done alongside a control group with hCG-enriched (25 mIU/ml) media only. For each set of experiments, all groups were done in duplicate on the same cell line (n = 2). XTT assays were further done in duplicate on the culture plates in order to obtain an average reading for data analysis. Testosterone and progesterone concentrations were quantified via ELISA in duplicate, and the mean of these results were entered for data analysis. Each set of experiments (n = 2) was repeated 6 times, providing n = 12 samples for analysis for each variable investigated. Experimental groups were compared to the control groups for cell viability (%), and testosterone and progesterone concentrations (ng/ml).

### Chemicals

Cells were cultured using standard sterile cell culture techniques, maintained using Dulbecco’s Modified Eagle Medium F-12 (DMEM/F-12) (Gibco, Johannesburg, South Africa) with 5% Horse Serum (Gibco), 2.5% Fetal Bovine Serum (Gibco) and 1% Penicillin-Streptomycin (Sigma-Aldrich, St. Louis, MO, USA). Cells were cultured in 75 ml culture flasks and incubated at 37 °C with 5% CO_2_. When confluent, and ready for experimental preparations, cultured cells were detached using 0.25% Trypsin/EDTA (Gibco).

Recombinant TNFα, IL1β, IL6 and IL8 appropriate for mouse cell culture experiments was obtained from Sigma-Aldrich as a lyophilised powder. As per manufacturer’s instructions, powders were reconstituted to achieve a 100 μg/ml solution. This solution was further diluted at 1:10 with culture medium in order to achieve 10 μg/ml stock solutions for each cytokine. Aliquots of this stock solution were frozen at − 20 °C until use. For each experiment, four concentrations of each cytokine were used: 100 ng/ml, 10 ng/ml, 1 ng/ml and 0.1 ng/ml. These were selected based on previous in vitro studies [19–33] and early experimental exposure of the cytokines to TM3 Leydig cells.

Recombinant insulin appropriate for mouse cell culture experiments was obtained from Sigma-Aldrich as a lyophilised powder. As per manufacturer’s instructions, the powder was reconstituted in 0.01 M hydrochloric acid (HCl) to achieve a 25 mg/ml solution, which was further serially diluted with culture medium to achieve a stock solution of 20 ng/ml. Aliquots of this stock solution were frozen at − 20 °C until use. For each experiment, four concentrations of insulin were used: 10 ng/ml, 1 ng/ml, 0.1 ng/ml, 0.01 ng/ml. These concentrations are based on previous literature [34–36] and early experimental exposure to TM3 Leydig cells in the laboratory to determine ideal experimental ranges.

### Cell culture

For each experiment, approximately 5000 cells per well were seeded in 300 μl medium in sterile 96-well plates and cultured for 48 h. Medium was then removed, and cells further cultured in experimental medium at the experimental concentrations for a further 48 h in the presence of 25 mIU/ml hCG. The respective controls were exposed to culture medium in the presence of 25 mIU/ml hCG only. After 48 h of exposure to the experimental concentrations, the experiments were terminated. Cell viability and steroid hormone (testosterone and progesterone) concentrations were determined for analysis. All experiments were done in duplicate, and repeated six times for data analysis variables (n = 12).

### Cell viability and proliferation determination

Cell viability was assessed using the Cell Proliferation Kit II (XTT) (Roche, Illovo, Johannesburg, South Africa). Under sterile conditions, XTT labelling reagent and the electron coupling reagent were thawed at 37 °C, mixed thoroughly, and 1 μl electron coupling reagent was added to 50 μl XTT labelling reagent as per instructions. From this, 50 μl XTT labelling mixture was added to 100 μl experimental medium to achieve a final XTT concentration of 0.3 mg/ml. This was incubated for 6 h in the dark at 37 °C. Following incubation, the plate was read with an ELISA reader (Labtech, East Sussex, UK) at a wavelength of 450 nm. Cell viability was expressed as a percentage of XTT binding as compared to the hCG control group.

### Steroid hormone quantifications

Testosterone and progesterone concentrations were determined from experimental cell culture supernatant. The supernatant was removed as cell culture media from each experimental well (300 μl) and stored in Eppendorf vials at − 20 °C until assayed. The testosterone and progesterone ELISA kits (DRG International, Inc., Springfield, New Jersey, USA) were used to determine testosterone and progesterone concentrations as per manufacturer instructions. All assays were carried out in duplicate, with the mean value recorded as the hormone concentration. Final concentration was quantified based on the standard curve determined by the supplied standard concentrations for the assay.

### Statistical analysis

Statistical analysis was performed using the MedCalc software (Version 12.0; Mariakerke, Belgium). All experiments were done in duplicate, and repeated six times (n = 12). Data was tested for distribution using the Kolmogorov-Smirnov test. Parametric data (normal distribution) is expressed as mean ± standard deviation in standard bar graphs, and experimental groups compared to the control group using the student T-test. For XTT analysis, the non-parametric Mann-Whitney test for independent samples was used to determine statistical significance of the experimental outcomes compared to the control group. The ANOVA one-way analysis of variance and ANOVA repeated measures analysis of variance were used to determine trends across increasing experimental concentrations on testosterone and progesterone production. A *P*-value of < 0.05 was considered significant.

## Results

### Effects of TNFα, IL1β and IL6

**Fig. 1:**
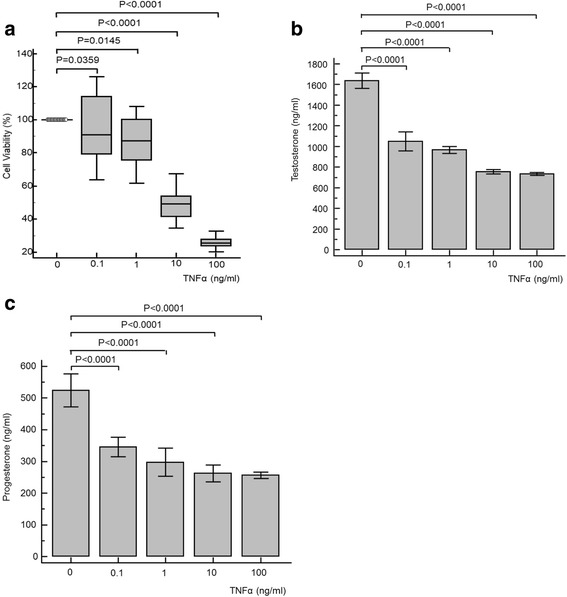
Cell viability (**a**), testosterone (**b**) and progesterone concentration (**c**) results for TM3 cell exposure to various concentrations of tumour necrosis factor-alpha (TNFα). All tested parameters significantly decreased for all concentrations of TNFα. For testosterone and progesterone results, ANOVA repeated measures analysis of variance was significant (*P* < 0.001), as was the one-way analysis of variance (*P* < 0.001)

**Fig. 2:**
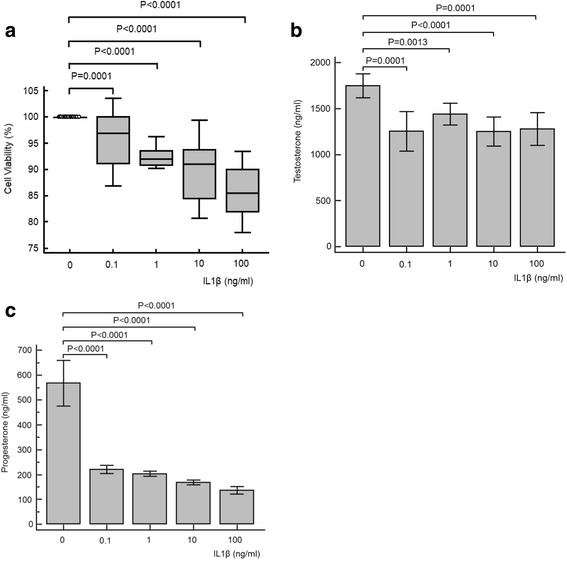
Cell viability (**a**), testosterone (**b**) and progesterone (**c**) results for TM3 cell exposure to various concentrations of interleukin 1-beta (IL1β). All parameters tested significantly decreased for all concentrations of IL1β. For testosterone, ANOVA repeated measures analysis of variance was not significant (*P* = 0.159), nor was the ANOVA one-way analysis of variance (*P* = 0.256). For progesterone, ANOVA repeated measures analysis of variance was significant (*P* < 0.001), as was the one-way analysis of variance (*P* < 0.001)

**Fig. 3:**
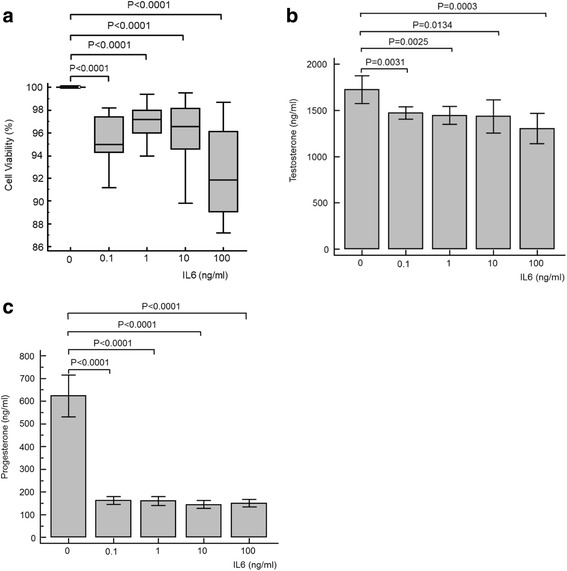
Cell viability (**a**), testosterone (**b**) and progesterone (**c**) results for TM3 cell exposure to various concentrations of interleukin 6 (IL6). All parameters significantly decreased for all concentrations of IL6. For testosterone and progesterone, ANOVA repeated measures analysis of variance was significant (*P* < 0.0001), as was the one-way analysis of variance (*P* < 0.001)

Exposure of TM3 Leydig cells to various concentrations of the inflammatory cytokines TNFα (Fig. 1), IL1β (Fig. 2) and IL6 (Fig. 3) resulted in significantly decreased cell viability, testosterone and progesterone concentrations compared to the control experiments. For TNFα and IL6, ANOVA repeated measures analysis and one-way ANOVA were significant (*P* < 0.001) for testosterone and progesterone concentrations, indicating a dose-dependent decline. For IL1β, there was no significant dose-dependent decrease in testosterone concentration observed. However, ANOVA repeated measures analysis and one-way ANOVA were significant (*P* < 0.001) for a dose-dependent decline in progesterone concentrations for IL1β.

### Effects of IL8

**Fig. 4:**
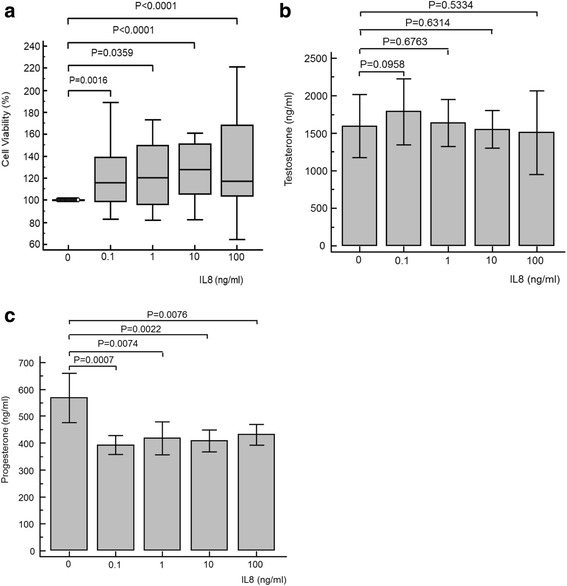
Cell viability (**a**), testosterone (**b**) and progesterone (**c**) results for TM3 cell exposure to various concentrations of interleukin 8 (IL8). Cell viability was significantly increased for all concentrations, while testosterone concentrations did not significantly change. Contrary, progesterone concentrations declined significantly. For testosterone, ANOVA repeated measures analysis of variance was not significant (*P* = 0.052), nor was the ANOVA one-way analysis of variance (*P* = 0.187). For progesterone, ANOVA repeated measures analysis of variance was significant (*P* = 0.002), as was the one-way analysis of variance (*P* < 0.001)

Exposure of TM3 Leydig cells to the cytokine IL8 resulted in a significant increase in cell viability compared to control for all concentrations (Fig. 4a). There was no effect of IL8 exposure observed on testosterone concentration, nor was there a significant dose-dependent relationship observed for testosterone concentration (Fig. 4b). However, an immediate and significant decrease in progesterone concentration was evident at all concentrations of IL8 investigated (Fig. 4c). Calculated over all IL8 concentrations applied, ANOVA repeated measures analysis (*P* = 0.002) and one-way ANOVA (*P* < 0.001) were significant for progesterone concentration (Fig. 4c).

### Effects of insulin

**Fig. 5:**
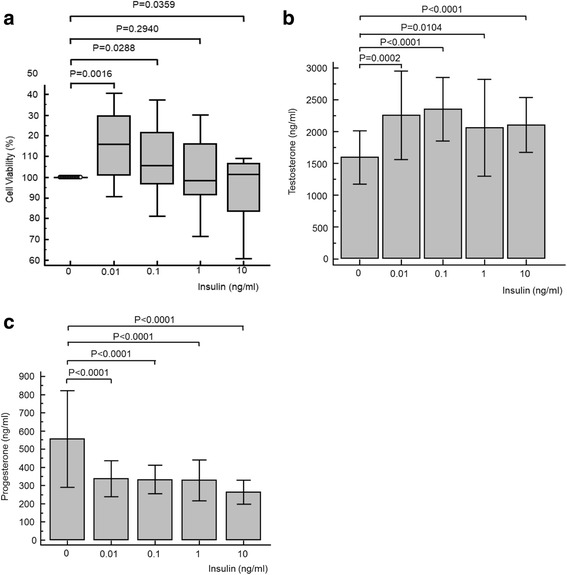
Cell viability (**a**), testosterone (**b**) and progesterone (**c**) results for TM3 cell exposure to various concentrations of insulin. Cell viability was significantly increased at 0.01, 0.1 and 1 ng/ml insulin. Testosterone concentrations were significantly increased for all concentrations, while progesterone concentrations significantly declined. For testosterone, ANOVA repeated measures analysis of variance was significant (*P* = 0.015), as was the one-way analysis of variance (*P* < 0.001). For progesterone, ANOVA repeated measures analysis of variance was statistically significant (*P* < 0.001), as was the one-way analysis of variance (*P* < 0.001)

Exposure of TM3 Leydig cells to insulin resulted in a significant increase in cell viability at 0.01 ng/ml (*P* = 0.002), 0.1 ng/ml (*P* = 0.029) and 10 ng/ml (*P* = 0.0359) (Fig. 5a). There was a significant increase in testosterone concentrations at all experimental concentrations tested, and increasing concentrations of insulin resulted in a dose-dependent and significant increase in testosterone production (ANOVA repeated measures analysis: *P* = 0.015; one-way ANOVA: *P* < 0.001) (Fig. 5b). However, the opposite was discovered for the progesterone production in which a significant decrease was observed at all experimental concentrations. Furthermore, there was a significant and dose-dependent decrease in progesterone concertation (ANOVA repeated measures analysis: *P* < 0.001; one-way ANOVA: *P* < 0.001) (Fig. 5c).

## Discussion

Lipophilic steroid hormones are produced via the enzymatic cascade termed steroidogenesis. This process can occur in various tissues (e.g. gonads, adrenal medula and adipose tissue) [13, 16], and testosterone synthesis in males is most prominent in the Leydig cells [16, 18]. Progesterone, although poorly studied, is an important precursor to testosterone and further has regulatory roles on male physiology and pathology [12]. This is mostly investigated on male contraception, the treatment of male hypersexuality and the role of progesterone in fertilisation [12]. In order to further elicit a possible causal relationship between inflammation and hyperinsulinaemia on modulating steroidogenesis, this study suggests that inflammatory cytokines negatively influence steroidogenesis cascades in TM3 Leydig cells, whereas insulin positively modulates testosterone production.

### Tumour necrosis factor-alpha

TNFα has been suggested to negatively affect the HPT axis, where in vivo administration is associated with a decrease in serum testosterone [1, 19]. The results of this study agree with previous publications that have assessed the impact of TNFα on Leydig cell function in vitro and in vivo [19–25]. TNFα reduces testosterone production in ranges of 0.1–100 ng/ml, typically in a dose dependant manner, in Leydig cells [20–24]. It is plausible that the enzymes mediating cholesterol uptake into the mitochondria (StAR), conversion to pregnenolone (P450_scc_), pregnenolone conversion to progesterone (3βHSD) and progesterone conversion to 17-OH-P (CYP17) are all negatively impacted by increasing TNFα concentrations [20–24]. Furthermore, the significant decrease in cell viability observed in this study would suggest cellular injury at increasing concentrations of TNFα exposure, leading to down-regulation of steroidogenesis enzyme production as a response to cellular injury [23]. TNFα was shown in this study to also decrease progesterone synthesis, agreeing agrees previous studies [20, 25] in Leydig cells. Based on the suggested mechanisms of steroidogenesis down-regulation [20–24], this would be an expected result that has been confirmed in this study.

### Interleukin 1-beta

Previous studies on the effect of IL1 on Leydig cell steroid regulation have provided conflicting results. This appears to be due to a variety of different stages of puberty in which primary Leydig cells were obtained from animals [26]. Similar to this study, IL1β has been shown to decrease hCG-stimulated testosterone synthesis in a dose dependant manner using concentrations of 1–100 ng/ml [27, 28]. This may be via a decrease in hCG stimulated cAMP production [27] and reduced P450_scc_ mRNA transcription [28]. In contrast, other studies have shown that IL1β did not significantly alter Leydig cell testosterone synthesis [29], or even increased testosterone synthesis [30].

Similar to this study, IL1 exposure has also previously been associated with a decrease in progesterone levels [25]. However, in contrast to this study, Wu and colleagues reported that a significant decrease in cell viability in TM3 Leydig cells exposed to 0.02 pg/ml IL1β which was interestingly prevented by co-stimulation of Sirt1, associated with cellular protection from inflammatory stress [23].

### Interleukin 6

The effect of IL6 on Leydig cell function and steroidogenesis has been less well studied in controlled experiments compared to TNFα and IL1β [3]. However, the available reports generally agree with the results of this study. Human application of subcutaneous single doses of IL6 causes significant short term decreases in testosterone levels with increases in LH [31], most marked at 3 μg/Kg [31]. It is also suggested that IL6 compromises cAMP induced expression of CYP17 and, interestingly, 17β-HSD, at concentrations of 100 ng/ml in primary mouse Leydig cells [3], resulting in reduced testosterone synthesis [31, 32]. IL6 also decreases CYP17 and 3βHSD expression [32]. IL6 reportedly reduces Leydig cell viability at 0.02 pg/ml [23] similarly to this study. Interestingly, in adrenal cells from older adult males, IL6 is suggested to increase aldosterone, cortisol and DHEA synthesis [33], up-regulating adrenal steroidogenesis which is predominantly focused on mineralocorticoids and glucocorticoids synthesis [1].

### Interleukin 8

Unlike TNFα, IL1β and IL6, there appear to be no published investigations into the possible effects of IL8 on steroidogenesis in Leydig cells. In this study, testosterone concentrations are maintained, but progesterone concentrations significantly decreased. It therefore appears that IL8 may influence progesterone via CYP17 or P450_scc_ or StAR. As testosterone is not different from controls over 48 h, this would imply that the Δ^5^-steroid pathway is at least maintained, for which CYP17 or P450_scc_ and StAR are required.

### Insulin

The results from this study agrees with reports in which insulin increases testosterone synthesis in the absence of insulin resistance [4, 34–38]. Exposure of primary mouse Leydig cells to 1 μg/ml insulin showed that for one hour prior to addition of LH for a further 3 h increased testosterone production compared to no insulin pre-treatment [34]. Lin et al. [36] also demonstrated insulin stimulation of testosterone synthesis in a primary rat Leydig cell culture model. Furthermore, 24-h exposure of primary catfish Leydig cells to 1 ng/ml insulin stimulated testosterone synthesis [39]. However, Ahn and colleagues [40] demonstrated that insulin binds directly to insulin receptors on the Leydig cell membrane, leading to an upregulation of DAX-1 genes expression via the AKT pathway in MA-10 Leydig cells. This in turn resulted in a downregulation of steroidogenesis in these cells [40], which is somewhat contrary to the testosterone results in this study and others [4, 34–38].

Insulin mediates metabolic and mitogenic effects through binding to cell surface insulin receptors, leading to activation of two pathways: the phosphoinositide 3-kinase (PI3K) and the mitogen-activated protein kinase (MAPK) pathways. PI3K results in activation of 3-phosphoinositide-dependent protein kinase 1 (PDK1) and Akt kinase, mediating most of the cellular effects of insulin [41]. The activation of the MAPK pathway generally mediates transcription of factors involved with cell growth. Effects of metabolites in this pathway result in vasoconstriction, increased expression of vascular cell adhesion molecules and growth and mitogenesis of vascular smooth muscle cells [41].

In animals and humans, hyperinsulinaemia and insulin resistance is closely associated with male hypogonadism [37, 42], which also conflicts with these in vivo results. In various cell lines (e.g. hepatocytes, adipocytes, skeletal muscle), the PI3K/Akt intracellular pathway through which insulin exerts its effects breakdown with increasing insulin resistance (hyperinsulinaemia) [41]. The induction of hyperinsulinaemia in mice results in disruption of steroidogenesis cascades via abnormal activation of the oestrogen receptor-alpha pathway, contributing to male hypogonadism pathogenesis [43]. Currently, no significant data exists in which to further postulate on the direct potential impact of hyperinsulinaemia and insulin resistance on Leydig cell function and steroidogenesis molecular mechanisms. Further research is required in order to further elicit these mechanisms.

There are no significant studies identified that investigate the effect of insulin on progesterone synthesis. In stark contrast to testosterone concentrations, progesterone was significantly decreased for all concentrations assessed, in a dose-dependent manner. As progesterone is an essential precursor of testosterone, these results suggest enzymatic activity downstream of progesterone may be upregulated, and not those upstream. Hypothetically, insulin may stimulate an upregulation of testosterone synthesis via the Δ^4^-steroid pathway, acting on one or more of these enzymes such as CYP17, CYP17,20 or 17β-HSD. This may also be associated with a down-regulate 3β-HSD, leading to a decrease in the Δ^5^-steroid pathway [44]. Further studies on these mechanisms is therefore warranted to further elicit the physiological and pathological mechanisms associated with insulin and Leydig cell steroidogenesis cascades.

### Clinical implications

Adequate Leydig cell function with the central purpose of testosterone production via steroidogenesis cascades is critical not only for male reproduction, but in general male health and well-being [6]. Various cytokines, as well as insulin, have been suggested to modulate the HPT axis, and may further regulate steroidogenesis within Leydig cells directly or indirectly [1, 3, 4]. Obesity and related metabolic co-morbidities and complications are closely associated with a low grade, asymptomatic, systemic and chronic inflammatory state and hyperinsulinaemia, amongst other underlying mechanisms [14, 15]. This is also relevant in male ageing and androgen decline in ageing men, which is associated with increasing inflammatory markers and hyperinsulinaemia [45]. Furthermore, increased systemic inflammatory cytokines and insulin have correlated with increased seminal concentrations of cytokines and insulin [14, 15]. As testosterone is critical in male health and well-being [5, 6], the (patho)physiological role of cytokines and insulin is relevant within the context of inflammation in males.

The T_H_1-lymphocyte associated inflammatory cytokines, specifically TNFα, IL1β, and IL6, are increasingly associated with male hypogonadism. Although a direct causal association is postulated, this is poorly understood [5, 9]. This is observed in acute inflammatory (infectious) disease associated with gonadal failure [5], and low-grade chronic inflammation associated with obesity and related co-morbidities and complications such as metabolic syndrome and type 2 diabetes mellitus [15]. Testosterone is also inversely correlated to the inflammatory marker C-Reactive Protein (CRP) in males, and males in lower quintiles of serum testosterone concentrations are more likely to have increased inflammatory markers associated with TNFα and IL6 [17]. In addition to having immune (down)regulating functions [17], testosterone therapy reduces inflammatory cytokine production and improves insulin sensitivity in obese and metabolic syndrome males [46].

Although poorly researched in males, reduced serum progesterone is associated with obesity and metabolic syndrome [44, 47, 48]. This study suggests that an associated pro-inflammatory state is associated with reduced progesterone as observed in observational clinical results. However, further research, particularly to understand the mechanisms associated with these results, is required in order to identify potentially novel therapeutic targets in males with chronic inflammation.

Insulin independently stimulates testosterone production and simultaneously inhibits SHBG in normal weight and obese males [4]. Furthermore, insulin resistance is closely associated with low testosterone in males [14, 42]. There is a close relationship between insulin sensitivity and testosterone concentrations in men across a wide range of glucose intolerance, including those with T2DM, and independent of SHBG concentrations [49]. This study is consistent with in vivo evidence from animals and humans that insulin increased testosterone production in insulin sensitive males [49]. However, insulin sensitivity is directly related to testosterone synthesis [34, 36]. Insulin appears to activate the Akt kinase pathway within Leydig cells, which likely mediates the role of this hormone in steroidogenesis [40]. It is further known that this intracellular pathway breaks down with insulin resistance in other cell lines [41]. Numerous studies have shown that TNFα can impair insulin signalling in hepatocytes, adipose tissue and skeletal muscle [50]. It is therefore feasible that induction of insulin resistance may result in the inability of insulin to promote testosterone synthesis in Leydig cells, and further contribute to hypogonadism. Further studies to understand the mechanism of insulin, including the potential induction of insulin resistance, on Leydig cell function and steroidogenesis required.

### Strengths and limitations of the study

This in vitro study was done under standard and controlled laboratory conditions. Experiments on each exposure were done in duplicate and assays further done on duplicate to ensure reliability of the results obtained. Experiments were replicated six times, and statistical analysis on the data is appropriate. This study is limited specifically in the number of variables investigated, due to the exploratory nature and aims of the study. Caution should be made in the interpretation of the results within the context of the existing literature, as different in vitro and in vivo models and methods are used in the studies reported. Implications of the results within the broader clinical context, although relevant, require further investigation, and these results are not able to directly prove causation relevant to clinical practice.

## Conclusions

The inflammatory cytokines TNFα, IL1β and IL6 cause a dose dependent decline in steroidogenesis in TM3 Leydig cells over 48 h. These results suggest that chronic inflammation may downregulate steroidogenesis in males via direct modulation of Leydig cell function. However, IL8 appears to minimally affect testosterone production in the concentrations observed, with an increase in cell viability and decrease in progesterone concentrations which may suggest inhibition of progesterone synthesis from cholesterol via 17-OH pregnenolone. Insulin was observed to have a dose-dependent increase in testosterone synthesis alongside a significant decline in progesterone synthesis, indicating insulin may directly act on Leydig cells as part of steroid hormone regulation. With the phenomenon of insulin resistance, the literature is unclear on the potential role of hyperinsulinaemia in steroidogenesis. Further studies are warranted in order to fully elicit the molecular mechanisms and interactions of these molecules on male steroidogenesis.

## Abbreviations

17β-HSD: 17β-Hydroxysteroid Dehydrogenase
3β-HSD: 3β-Hydroxysteroid Dehydrogenase
Akt kinase: Protein Kinase B
cAMP: Cyclic Adenosine Monophosphate
CYP17: Cytochrome P450 17α-Hydroxylase
CYP17,20: Cytochrome P450 17,20-Lyase
DHEA: Dehydroepiandrosterone
EDTA: Ethylenediaminetetraacetic acid
FSH: Follicular Stimulating Hormone
GnRH: Gonadotropin releasing hormone
hCG: Human Chorionic Gonadotropin
HPT: Hypothalamic-Pituitary-Testes Axis
IL1β: Interleukin 1-beta
IL6: Interleukin 6
IL8: Interleukin 8
LH: Luteinizing Hormone
NO: Nitric Oxide
P450_scc_: Cholesterol Side-Chain Cleavage Enzyme
SHBG: Sex Hormone Binding Globulin
Sirt1: Sirtuin 1
StAR: Steroidogenic Acute Regulatory Protein
TNFα: Tumour Necrosis Factor-Alpha
